# Nascent transcription reveals regulatory changes in extremophile fishes inhabiting hydrogen sulfide-rich environments

**DOI:** 10.1098/rspb.2024.0412

**Published:** 2024-06-19

**Authors:** Blair W. Perry, Kerry L. McGowan, Lenin Arias-Rodriguez, Sascha H. Duttke, Michael Tobler, Joanna L. Kelley

**Affiliations:** ^1^ School of Biological Sciences, Washington State University, Pullman, WA 99164, USA; ^2^ School of Molecular Biosciences, Washington State University, Pullman, WA 99164, USA; ^3^ División Académica de Ciencias Biológicas, Universidad Juárez Autónoma de Tabasco, Villahermosa, Tabasco 86150, México; ^4^ Department of Biology, University of Missouri—St Louis, St Louis, MO 63121, USA; ^5^ Whitney R. Harris World Ecology Center, University of Missouri—St Louis, St Louis, MO 63121, USA; ^6^ WildCare Institute, Saint Louis Zoo, St Louis, MO 63110, USA; ^7^ Department of Ecology and Evolutionary Biology, University of California Santa Cruz, Santa Cruz, CA 95060, USA

**Keywords:** capped-small RNA-sequencing, transcriptomics, transcription factor, *Poecilia mexicana*, adaptation

## Abstract

Regulating transcription allows organisms to respond to their environment, both within a single generation (plasticity) and across generations (adaptation). We examined transcriptional differences in gill tissues of fishes in the *Poecilia mexicana* species complex (family Poeciliidae), which have colonized toxic springs rich in hydrogen sulfide (H_2_S) in southern Mexico. There are gene expression differences between sulfidic and non-sulfidic populations, yet regulatory mechanisms mediating this gene expression variation remain poorly studied. We combined capped-small RNA sequencing (csRNA-seq), which captures actively transcribed (i.e. nascent) transcripts, and messenger RNA sequencing (mRNA-seq) to examine how variation in transcription, enhancer activity, and associated transcription factor binding sites may facilitate adaptation to extreme environments. csRNA-seq revealed thousands of differentially initiated transcripts between sulfidic and non-sulfidic populations, many of which are involved in H_2_S detoxification and response. Analyses of transcription factor binding sites in promoter and putative enhancer csRNA-seq peaks identified a suite of transcription factors likely involved in regulating H_2_S-specific shifts in gene expression, including several key transcription factors known to respond to hypoxia. Our findings uncover a complex interplay of regulatory processes that reflect the divergence of extremophile populations of *P. mexicana* from their non-sulfidic ancestors and suggest shared responses among evolutionarily independent lineages.

## 1. Introduction

Changes in gene expression can facilitate the alteration of phenotypes. There are many examples of transcriptional differences underlying dramatic phenotypic changes, ranging from tissue regeneration [1] and diapause [2] to stress responses [3]. While evolutionary biologists have long focused on nucleotide changes in protein-coding genes to explain phenotypic evolution [4,5], foundational work has also shown that changes in the expression of protein-coding genes can drive evolutionary change and mediate adaptation [6–8]. Well known examples of adaptive gene expression changes include high-altitude muscle function [9] and coat camouflage for predator avoidance [10]. However, the regulatory mechanisms that drive changes in gene expression are often complex and difficult to study, and therefore remain poorly understood. A better understanding of gene regulatory networks and the molecular mechanisms underlying changes in gene expression is critical to help explain patterns of adaptation and plasticity [11].

Gene expression is largely regulated by activity in non-coding genomic regions and often involves the transcription of different types of RNA, including microRNAs (miRNAs) [12], RNA transcripts from promoter, terminal and enhancer regions [13,14] and long non-coding RNAs [15]. Additionally, the binding of transcription factor (TF) proteins in promoters and enhancers initiates and regulates transcription, such that changes in TF binding sites and TF activity can have dramatic effects on gene expression and ultimately emerging phenotypes [16,17]. Understanding the impact of regulatory activity is therefore key to explaining how responses to an environment occur at the genomic level, and how they might evolve.

**Figure 1.**
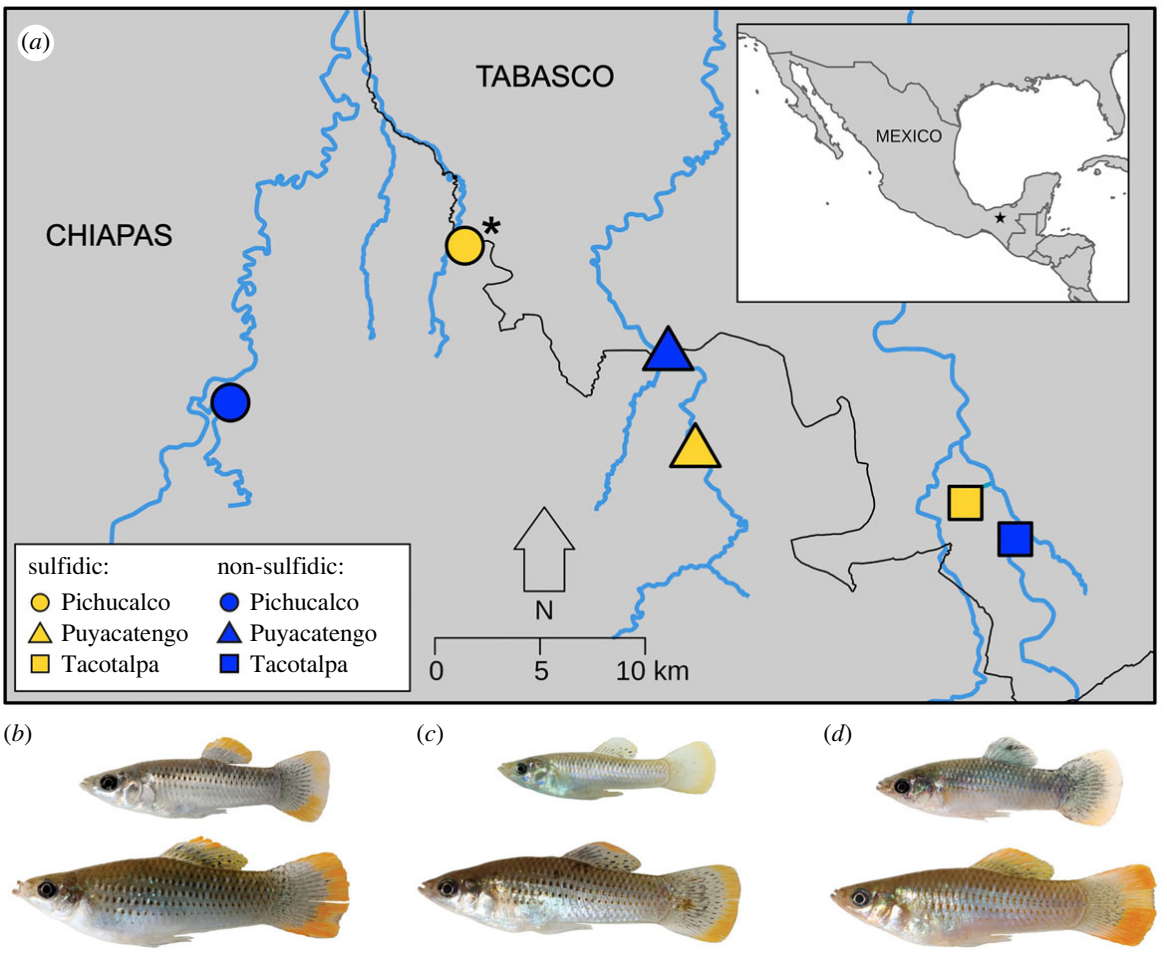
(*a*) Map of sampling sites in the Pichucalco, Puyacatengo and Tacotalpa drainages in the Río Grijalva basin in southern Mexico. Shapes indicate drainage of origin. Colours indicate whether sites were sulfidic (yellow) or non-sulfidic (blue). In the Pichucalco drainages, sulfidic stream sites (marked with *) are inhabited by the endemic *Poecilia sulphuraria* species, while the non-sulfidic streams in this drainage are inhabited by *Poecilia mexicana*. Both sulfidic and non-sulfidic streams in the Puyacatengo and Tacotalpa drainages are inhabited by *P. mexicana*. (*b–d*) Fish sampled from sulfidic (top) and non-sulfidic (bottom) streams in the (*b*) Pichucalco, (*c*) Puyacatengo and (*d*) Tacotalpa drainages. Fish not to scale. Photo credit: Michael Tobler.

Species in which variation in gene expression has been linked to adaptation offer opportunities to study mechanisms of gene regulation. Fishes in the *Poecilia mexicana* (Atlantic molly) species complex (family Poeciliidae) are one such example. *Poecilia mexicana* is a livebearing fish common in freshwater habitats throughout Mexico and Central America [18]. In southern Mexico, multiple lineages have independently colonized toxic springs rich in H_2_S (figure 1*a*; [19]). Sulfide spring populations (i.e. sulfidic ecotypes) are locally adapted and are phenotypically and genetically distinct from adjacent populations in non-sulfidic habitats [20–22]. H_2_S is toxic to most animals, inhibiting enzymes in the electron transport chain and lethally interfering with aerobic ATP production via oxidative phosphorylation [23,24]. H_2_S also reacts with dissolved oxygen in aquatic environments, creating additionally stressful hypoxic conditions [25,26]. The ability to modulate gene expression plays a major role in survival in these H_2_S-rich environments, with sulfidic populations showing convergent shifts in the expression of key genes involved in H_2_S detoxification and sulfur metabolism [21,22,27,28]. Many of these changes involve genes in the sulfide : quinone oxidoreductase (SQR) pathway, which endogenously detoxifies H_2_S [29], This pathway is present in all metazoans and likely a bacterial relic from the Proterozoic aeon [30]. The expression of genes involved in the SQR pathway is consistently upregulated in sulfidic populations, as are sulfur transporter genes, and genes encoding subunits of cytochrome *c* oxidase (COX, the main toxicity target of H_2_S) [21,22,27,28]. Some of these genes show evolved, constitutive expression differences in sulfidic populations compared with non-sulfidic populations, whereas other genes show evolved plasticity in sulfidic populations in response to H_2_S [31]. However, aside from some evidence that miRNAs and DNA methylation changes affect the expression levels of many H_2_S-related genes in one population [32,33], the underlying regulatory processes responsible for the changes in gene expression observed in this system remain underexplored. Examining these mechanisms will provide a better understanding of the regulatory changes underlying parallel adaptation in these fishes.

To examine regulatory differences among populations in the *P. mexicana* species complex, we used capped-small RNA-sequencing (csRNA-seq), a nascent RNA sequencing method that captures short, newly initiated transcripts (approx. 20–60 nucleotides in length) [34]. In contrast to traditional RNA-sequencing approaches (i.e. RNA-sequencing with either messenger RNA (mRNA) or ribosomal depletion methods) which capture only the mature, stable transcripts of genes, nascent RNA-sequencing methods like csRNA-seq instead capture all initiated transcripts, including unstable transcripts [34–37]. These unstable RNAs (i.e. enhancer RNAs, miRNAs, antisense transcripts and other regulatory RNAs) persist for only a short time in the cell but can have important effects on gene regulation [38–40]. csRNA-seq can pinpoint transcriptional changes in stable and unstable regulatory RNAs and identify initiation sites in the genome to base-pair (bp) resolution [34]. Capturing this positional information and identifying shared changes in regulatory RNA transcription across independently adapted populations will reveal how regulatory mechanisms alter gene expression to enable survival of *P. mexicana* in sulfidic environments. Given that sulfidic lineages are often genetically distinct from adjacent non-sulfidic populations [20–22], shared differences across independent lineages may be indicative of adaptation [41]. More broadly, this approach will provide valuable new insight into the mechanisms by which gene expression regulation may evolve in response selection.

In this study, we sampled three population pairs in the *P. mexicana* species complex comprising both ancestral non-sulfidic and corresponding H_2_S-adapted linages, and we investigated differences in patterns of nascent transcription between ecologically contrasting populations. Specifically, we asked the following questions: (1) What regulatory mechanisms underlie differences in gene expression in sulfidic populations compared with non-sulfidic populations? (2) What TF binding sites are overrepresented near differentially initiated transcription start sites of genes with gene expression shifts in sulfidic fish? (3) Are the genes coding for TFs with enriched binding sites differentially expressed between ecotypes? (4) Are differences in gene expression between ecotypes driven in part by differential activity of enhancer regions? To answer these questions, we first investigated variation across two distinct aspects of gene expression. In addition to capturing variation in transcript initiation with csRNA-seq, we also quantified shifts in gene expression using mature, stable mRNAs from coding regions using previously published RNA-sequencing (mRNA-seq) data [27]. We identified unique patterns of transcript initiation in sulfidic populations that were associated with H_2_S detoxification and identified TFs that may be particularly important in controlling gene expression in sulfidic environments. These analyses highlight regulatory variation between H_2_S-adapted populations and ancestral non-sulfidic populations and shed light on regulatory mechanisms that underlie changes to some of the most conserved metabolic pathways in metazoans, including H_2_S detoxification and oxidative phosphorylation.

## 2. Methods

### (a) Sample collection

Adult female fish were collected via seine net from sulfidic and non-sulfidic sites in the Pichucalco, Puyacatengo and Tacotalpa drainages of the Río Grijalva basin in southern Mexico (*N* = 5 or 6 per site; electronic supplementary material, table S1) as part of a previous study by Kelley *et al.* [27]. In the Pichucalco drainage, sulfidic springs are inhabited by the highly endemic extremophile *Poecilia sulphuraria* (sulphur molly; figure 1*b*, top) [42], but non-sulfidic springs are populated by *P. mexicana* (figure 1*b*, bottom). In the Puyacatengo and Tacotalpa drainages, *P. mexicana* inhabits both the sulfidic and non-sulfidic sites (figure 1*c,d*). Fish were sacrificed, and their gill arches were extracted, preserved in RNAlater (Invitrogen), and stored at −80°C. Gill tissues were selected because they are directly exposed to H_2_S in the water and were previously shown to have significant gene expression differences between the sulfidic and non-sulfidic ecotypes [27,31].

While inter-drainage variation in adaptation to H_2_S has been demonstrated in *Poecilia* [4,22], we were most interested in the broad patterns of gene regulation across ecotypes, as there is pre-established convergence in the differential expression of H_2_S-related genes in all sulfidic populations [27,31]. For this reason, we compared gill tissues sampled from all sulfidic fish with those from all non-sulfidic fish combined across drainages.

### (b) csRNA library preparation and sequencing

csRNA-seq was generated from a subset of the fish samples described above (*N* = 2 fish per site; electronic supplementary material, table S1). Total RNA was extracted from gill tissues using Qiagen's miRNeasy Mini Kit following all standard protocols. Total RNA concentrations were estimated with the Qubit RNA HS Assay Kit and the Agilent 2100 BioAnalyzer using the RNA 6000 Nano Kit. Short RNAs (approx. 20–60 nucleotides) were isolated using size selection with gel electrophoresis. For each sample, a 10% aliquot of the size-selected RNA suspension was reserved for use as an ‘input library’ [43]. These aliquots were sequenced and used in the HOMER pipeline to control for exonic contamination when identifying transcription initiation sites [34]. For the remaining size-selected RNA for each sample, 5′ 7-methylguanosine- capped RNA was isolated following protocols from Duttke *et al*. [34], and this constituted our csRNA libraries. csRNA and the reserved input aliquots were converted into cDNA libraries for each sample, amplified with PCR, size-selected to remove primer dimers, purified with gel electrophoresis and pooled, and single-end 75 bp reads were sequenced using an Illumina NextSeq 500.

### (c) Analysis of differential transcription initiation using csRNA-seq

We used FastQC (v. 0.11.9) to examine the quality of the raw csRNA and input RNA reads [44]. Subsequent analyses were carried out using the csRNA-seq analysis pipeline in HOMER (v. 4.11) [34]. We used HOMER's *trim* to remove Illumina TruSeq adapters from the 3′ end of reads, discarded reads less than 20 bp, and trimmed the ends of reads below a Phred score of 20. We indexed the *P. mexicana* reference genome (GenBank assembly accession: GCF_001443325.1) [45] using STAR (v. 2.7.6a) and then aligned the reads with STAR, outputting one primary alignment per read [46]. Tag directories were created for the uniquely mapped csRNA libraries and the input libraries for each sample using HOMER's *makeTagDirectory*, including the *--single* flag to account for the large number of scaffolds in the reference genome and *--fragLength* set to 30 bp to reflect the average length of the nascent RNAs captured by the csRNA-seq protocol [34]. A third tag directory was created for each sample using the mapped mRNA libraries (described below) and used to control for false positives and exonic contaminants in the next step. The csRNA, input and mRNA tag directories were input into HOMER's *findcsRNATSS.pl* to identify sites of transcription initiation, hereafter called peaks. This step of the HOMER pipeline also determines the stability of initiated transcripts by assessing whether stable mRNA transcripts map within −100 to +500 bp of each csRNA-seq peak. Unstable, nascent transcripts are not processed into stable mRNAs and are thus determined by the lack of stable mRNA transcripts mapping to the adjacent region (see [34] for additional detail). We retained peaks with at least seven csRNA-sequencing reads per 10 million reads for further analysis following the default parameters. Peaks were annotated using the GTF file for *P. mexicana* (GenBank assembly accession: GCF_001443325.1, converted from GFF using *gffread* (v. 0.9.9)). HOMER's *mergePeaks* was used to create a non-redundant list of peaks across all samples, condensing peaks that directly overlapped. The merged peaks file was used with homerTools *annotatePeaks.pl* and the *--strand + --fragLength 1* options required for csRNA-seq to quantify the raw read counts for each sample, associate each peak with the nearest annotated gene from the GFF, and identify the region of the genome they mapped to relative to this gene (i.e. in an exon, intergenic, intron, promoter or transcription start site (promoter-TSS), or transcription termination site (TTS)). The promoter-TSS region is defined as the region 1000 bp upstream to 100 bp downstream of a gene's annotated TSS in the GTF file. We the identified differentially initiated peaks in R using edgeR (v. 4.0.12) [47] by comparing all sulfidic samples with all non-sulfidic samples while including drainage and species as covariates. Significantly differentially initiated peaks were defined as those with a false discovery rate (FDR) <0.05. Variation in transcription initiation across samples was plotted using a principal components analysis (PCA), using the 500 peaks with the highest average signal across all samples using the R package *limma* (v. 3.46.0). Hierarchically clustered heatmaps of csRNA-seq peaks were generated with the R package *pheatmap* (v. b1.0.12) using the top 5000 peaks based on overall number of counts across samples and subsequently with only significantly differentially initiated peaks.

We used a curated list of genes with Gene Ontology (GO) terms related to H_2_S detoxification or response (GO:0070221, GO:0006790, GO:0044273, GO:0070813, GO:0000096, GO:0006090, GO:0006749, GO:1901687, GO:0000098, GO:0008272, GO:0006534, GO:0044272) to carry out a Fisher's exact test using *fisher.test()* in base R (v. 4.0.3), which tested whether peaks in or near genes related to H_2_S detoxification and response were overrepresented in the list of significantly differentially initiated peaks.

### (d) mRNA library preparation and sequencing

Samples were prepared and sequenced as part of a previous study by Kelley *et al.* [27]. In brief, total RNA was extracted from gill tissues (*N* = 17 sulfidic fish, *N* = 18 non-sulfidic fish; electronic supplementary material, table S1), poly-A mRNA was isolated, and libraries were sequenced using an Illumina HiSeq 2000 using paired-end 101 bp reads.

### (e) mRNA differential gene expression

The quality of all raw reads was assessed using FastQC. Trim Galore! (v. 0.4.2) was used to remove the first 11 bp from the 5′ ends of reads (*--clip_R1 11 --clip_R2 11*) and perform adapter trimming (*--stringency 6*) but not quality trimming (*--quality 0*) [48]. A second round of Trim Galore! was used to quality trim the ends of reads with a Phred score below 24 (*--quality 24*) and remove reads less than 50 bp long (*--length 50*). HISAT2 *hisat2-build* (v. 2.1.0) was used to index the *P. mexicana* reference genome with the mitochondrial genome appended (GenBank accession: KC992995.1) [4]. Trimmed reads were mapped using the *--downstream-transcriptome-assembly* flag for compatibility with StringTie (v. 2.0.3), *--fr* specifying the mate pair orientation, and read group information included for sample, read group identifier and platform [49]. SAMtools *view* (v. 1.9) was used to convert the resulting SAM files to BAM files, *sort* to sort the BAM files by coordinate, and *merge* to combine samples with more than one technical replicate [50]. StringTie (v. 2.0.3) with the *--eB* flag was used to generate read coverage tables in GTF format for each sample [51]. An edited version of the prepDE.py script from StringTie (see code repository) was used to generate gene count matrices, resulting in counts for nuclear genes only. Genes were filtered by a minimum counts-per-million (cpm) of at least 5 in the smallest library and to have non-zero counts in at least five samples. Using edgeR (v. 4.0.12), libraries were normalized using the trimmed mean of *M*-values (TMM, the default) and quasi-likelihood *F*-tests were used to compare differential gene expression in sulfidic samples compared with non-sulfidic samples, with species and drainage included as covariates [47,52].

### (f) Nascent transcription near regions with stable gene expression

We investigated if patterns in differential transcript initiation between ecotypes matched patterns in nearby gene expression. Peaks with differential transcript initiation from csRNA-seq (FDR < 0.05) were intersected with differentially expressed genes from mRNA-seq (FDR < 0.05) based on their gene annotation to identify genomic regions that showed both changes in nascent transcription and stable gene expression between ecotypes. We then examined directionality to see if csRNA and mRNA datasets were both upregulated in sulfidic samples compared with non-sulfidic samples, both downregulated, or divergent. Those that were upregulated in both datasets or downregulated in both datasets are hereafter referred to as the ‘parallel upregulated subset’ and ‘parallel downregulated subset’, respectively.

### (g) Transcription factor binding site enrichment in differentially initiated promoter regions

We identified TF binding sites in differentially initiated csRNA-seq peaks in the promoter-TSS regions of differentially expressed genes, focusing specifically on the upregulated and downregulated subsets defined above. We used homerTools *findMotifsGenome.pl* to extract FASTA sequences spanning a 200 bp region from 150 bp upstream through 50 bp downstream of the peak centre, which should approximate the site of transcription initiation. We chose this narrow window in order to conservatively identify only the primary TF binding sites involved in initiating gene expression. We used CiiiDER [53] to identify significantly enriched TF binding motifs in these regions using the JASPAR CORE 2022 non-redundant vertebrate transcription factors database [54]. We used a maximum deficit of 0.15 to allow for non-exact motif matching, and used a stringent *p*-value cutoff of 0.01 for classifying significantly enriched binding sites. A set of promoter-TSS regions that were not differentially initiated (FDR > 0.05 and log_2_ fold-change < 0.5) and were not associated with a differentially expressed gene between ecotypes was used as the background for these enrichment analyses (*n* = 7443).

### (h) Identification and analysis of putative enhancer regions

csRNA-sequencing can capture enhancer RNAs, small unstable transcripts that result from active enhancer regions [55,56]. Enhancer RNA abundance often correlates with the expression of an enhancer's target gene(s) [57,58]; we therefore correlated differential initiation of putative enhancer csRNA-seq peaks with the expression of nearby genes to identify putative enhancers exhibiting differential activity between ecotypes and to infer genes that may be targeted by these enhancers. csRNA-seq peaks putatively corresponding to enhancer regions were defined as peaks annotated as distal from nearby genes (more than 500 bp from the promoter region of the nearest gene) and comprising unstable transcripts (i.e. transcripts present in csRNA but not mRNA libraries). This list of peaks was then intersected with the results of differential initiation analysis to identify putative enhancers with significantly increased or decreased activity in the sulfidic ecotype.

We then identified all significantly differentially expressed genes with a TSS located less than 500 kbp from the centre of an ecotype-responsive putative enhancer peak by first expanding all ecotype-responsive putative enhancer peaks by 500 kbp in both directions using the *slop* tool within bedtools (v. 2.30.0) and then intersecting these expanded regions with a bedfile of gene TSS positions using bedtools *intersect*. The resulting pairs of putative enhancers and potential target genes were filtered to retain those with the same direction of differential initiation/expression in the sulfidic ecotype (i.e. upregulated csRNA-seq peak and upregulated gene expression, and *vice versa*), and the log_2_ fold-change values of csRNA-seq and mRNA-seq were correlated using linear regressions in R. To focus downstream analyses only on putative target genes with high correlations to putative enhancer regions, pairs of putative enhancers and target genes were filtered to retain only those falling within one standardized residual of the regression line.

For the resulting putative enhancer peaks that were highly correlated with the expression of their inferred target gene(s), fasta sequences were extracted using the getfasta tool from bedtools. A background set of 1000 peaks were randomly selected from all putative enhancer peaks that were not differentially initiated between ecotypes (i.e. distal csRNA-seq peaks comprising unstable transcripts and with an FDR > 0.05 in differential initiation analyses). CiiiDER was again used to identify significantly enriched TF binding motifs in these regions using the JASPAR CORE 2022 non-redundant vertebrate transcription factors database. We used a maximum deficit of 0.15 to allow for non-exact motif matching, and used a stringent *p*-value cutoff of 0.01 for defining significantly enriched binding sites.

## 3. Results

### (a) Transcriptional differences across ecotypes

Capturing actively initiating RNAs with csRNA-seq in sulfidic and non-sulfidic populations (see electronic supplementary material, tables S2 and S3) identified an average of 27 021 ± 2096 regions of active transcription initiation (peaks) per sample (see electronic supplementary material, table S4 for per-population metrics). Merging all directly overlapped peaks resulted in a total of 66 537 non-redundant peaks across all samples.

**Figure 2.**
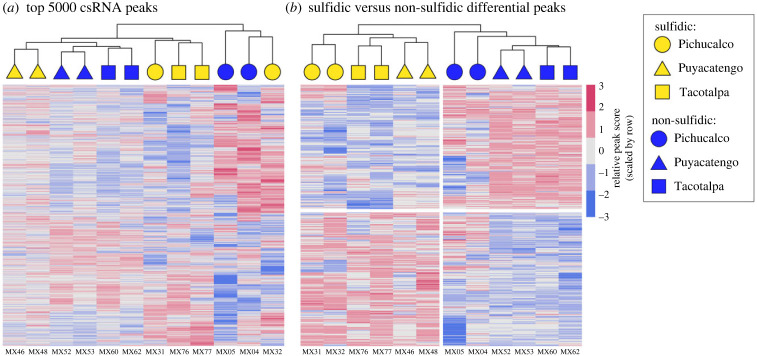
Hierarchical clustering analyses of (*a*) the top 5000 highest-abundance csRNA-seq peaks and (*b*) significantly differentially initiated peaks between sulfidic and non-sulfidic ecotypes. Each row represents a peak, each column a sample. The top dendrogram groups samples by similar patterns of transcript initiation. Heatmap colours represent relative peak score values, scaled by row to accentuate variation across samples. Shapes along the top dendrogram represent different drainages, with colour representing ecotype. Sample ID is listed below each column.

Hierarchical clustering of the top 5000 highest-scoring csRNA peaks grouped all samples by population except for one individual from the Pichucalco drainage (figure 2*a*). In a PCA generated from the top 5000 highest-scoring csRNA peaks, *P. sulphuraria* and *P. mexicana* populations separated along the second axis of variation, while the first axis of variation was driven largely by samples from the non-sulfidic Pichucalco population (electronic supplementary material, figure S1). Clustering of significantly differentially initiated peaks grouped samples within both ecotypes by population, indicating that population-specific variation in transcription initiation remains present even for peaks with broad differences in initiation between ecotypes (figure 2*b*).

We identified 1061 peaks with significantly different csRNA-seq peak density (i.e. differentially initiated peaks) between the two ecotypes (FDR < 0.05; electronic supplementary material, table S5). Of those, 550 peaks were upregulated and 511 peaks were downregulated in sulfidic samples compared with non-sulfidic samples. Peaks of transcription initiation located in or near genes involved in H_2_S detoxification and response were roughly twice as likely to be differentially active between ecotypes (odds ratio = 1.997, 95% CI = 0.974–3.688) as transcripts that were not related to H_2_S (two-sided Fisher's exact test, *p* < 0.05; electronic supplementary material, table S6). Three peaks upregulated in sulfidic populations mapped to upstream regions of the genes encoding sulfide : quinone oxidoreductase (*sqrdl*) and persulfide dioxygenase (*ethe1*), both of which are directly involved in H_2_S detoxification via the SQR pathway in the mitochondria (electronic supplementary material, table S6). Differential transcription initiation also occurred near the genes encoding a glutathione *S*-transferase (*gstz1*), chloride intracellular channel (*clic2*), and solute carrier (*slc16a6b*), among others (electronic supplementary material, table S6).

### (b) Differential gene expression across ecotypes

We compared gill mRNA-seq data from Kelley *et al.* [27] with ecotype-level differences in nascent transcription to examine the relationship between coding and non-coding transcription shifts in the sulfidic ecotype. We sequenced a total of 531 million mRNA reads, with an average of 15 ± 0.9 million reads per sample (see electronic supplementary material, tables S7 and S8 for read counts and mapping statistics per population). Gene expression clustered samples by ecotype along the first and second axes of variation in the PCA, with *P. sulphuraria* from the Pichucalco drainage clustering separately from the sulfidic *P. mexicana* from the Puyacatengo and Tacotalpa drainages (electronic supplementary material, figure S2). Analysis of differential expression between sulfidic and non-sulfidic ecotypes identified 2852 differentially expressed genes, 1395 of which were upregulated in the sulfidic ecotype (FDR < 0.05; electronic supplementary material, table S9).

### (c) Ecotype-level changes in nascent transcription paralleled those in gene expression

**Figure 3.**
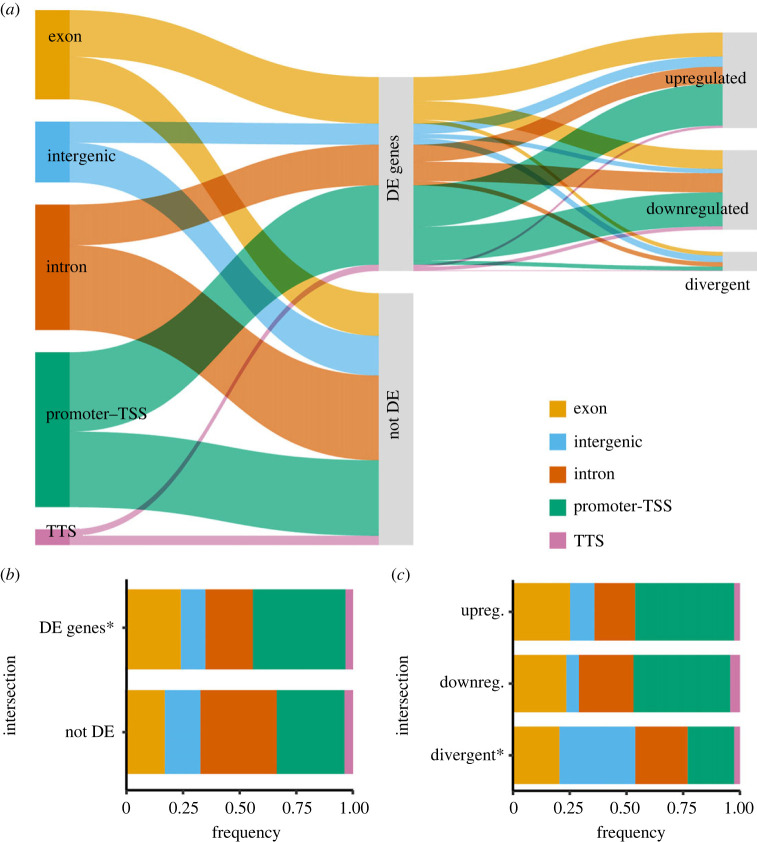
(*a*) The intersection of differentially initiated (DI) peaks (FDR < 0.05) from csRNA-seq with differentially expressed (DE) genes from mRNA-seq (FDR < 0.05). Of the DI peaks that also showed DE, most showed parallel shifts in the level of transcription (i.e. upregulated in both datasets, downregulated in both datasets) instead of divergent shifts. (*b*) Relative frequency of genomic locations of DI peaks associated with a DE gene (top bar) and DI peaks not associated with a DE gene (bottom bar). (*c*) Genomic locations of DI peaks with DE genes that were both upregulated (top bar), downregulated (middle bar), or divergent (bottom bar). TSS = transcription start site, TTS = transcription termination site. Asterisks indicate significantly different proportions in that group of peaks compared with all peaks combined (*G*-test goodness of fit tests, *p* < 0.05).

Most nascent transcription occurred near coding regions, particularly in the promoter-TSS regions (32.7%) and introns (28.1%; figure 3*a*). Of the 910 differentially initiated peaks that were annotated with a nearby gene, 44% were associated with genes found to be DE in analyses of mRNA-seq (figure 3*a*, ‘DE genes’).

The genomic annotations of csRNA peaks differed depending on whether the nearest gene was differentially expressed (figure 3*b*); csRNA peaks near differentially expressed genes were predominately located within promoter-TSS regions (figure 3*b*, DE genes), whereas peaks associated with non-differentially expressed genes were located primarily in intronic regions (figure 3*b*, not DE).

Of the 396 differentially initiated csRNA peaks associated with differentially expressed genes, 90% showed parallel shifts in the level of transcription (i.e. either both upregulated or both downregulated; figure 3*a*). We refer to these groups of peaks and associated genes as the ‘parallel upregulated subset’ and ‘parallel downregulated subset’ for simplicity. Peaks in these two parallel subsets were primarily located in promoter-TSS regions (figure 3*c*, ‘upregulated’ and ‘downregulated’), whereas peaks that showed an opposite change in magnitude to the nearby gene were predominately located in intergenic regions (figure 3*c*, 'divergent').

### (c) Transcription factor binding site enrichment reveals overrepresented motifs in sulfidic populations

**Figure 4.**
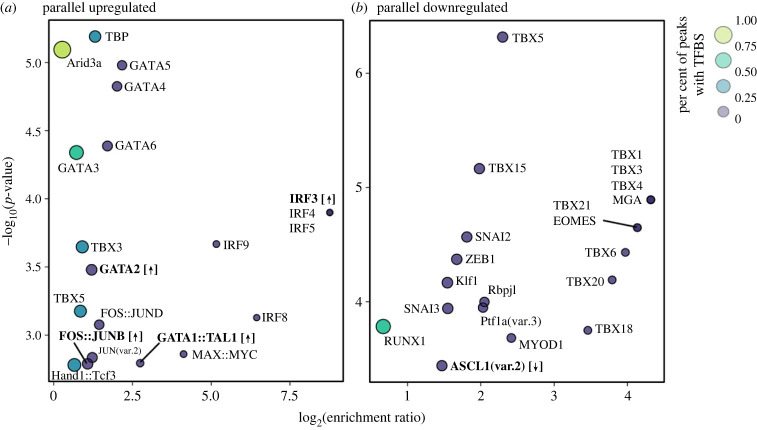
The top 20 most significantly enriched transcription factor binding sites (TFBSs) in (*a*) significantly upregulated csRNA peaks (csRNA-seq log_2_ fold-change > 0, FDR < 0.05) in the promoter-TSS of significantly upregulated genes (mRNA-seq log_2_ fold-change > 0, FDR < 0.05) and (*b*) significantly downregulated csRNA peaks (csRNA-seq log_2_ fold-change < 0, FDR < 0.05) in the promoter-TSS of significantly downregulated genes (mRNA-seq log_2_ fold-change < 0, FDR < 0.05). The log_2_ enrichment ratio is represented on the *x*-axis, with points to the right in each panel having a higher degree of overrepresentation in the target peak set compared with the background. The −log_10_(*p*-value) of the enrichment test is represented on the *y*-axis, with points towards the top of each panel having lower *p*-values. Point size and colour are scaled by the proportion of target peaks possessing a given TFBS. Bold labels indicate TFBSs for which the gene encoding that TF is significantly upregulated (up arrows) or downregulated (down arrow) in the sulfidic ecotype. The '::' separates co-binding TFs. For a list of all enriched TFBSs, see electronic supplementary material, table S9.

To investigate TFs with potential roles in driving ecotype-specific gene expression, we performed TF binding site enrichment analysis in all promoter-TSS peaks belonging to the parallel upregulated and parallel downregulated subsets defined above (85 peaks in the parallel upregulated subset, 69 in the parallel downregulated subset). We identified 65 TF binding sites that were significantly enriched in the parallel upregulated promoter-TSS peaks and 56 enriched in the parallel downregulated promoter-TSS peak set, with 10 TF binding sites found to be enriched in upregulated and downregulated promoter-TSS peaks (*p* < 0.01, figure 4; electronic supplementary material, table S10). Of the 65 TF binding sites enriched in the parallel upregulated peak set, nine were further supported by significant upregulation of a gene encoding the TF (GATA2, SP8, IRF3, STAT::STAT2, GATA1::TAL1, KLF11, FOSB::JUNB, SHOX and Mafb; figure 4; electronic supplementary material, table S11). Only three TFs with enriched binding sites in the parallel downregulated peak set were further supported by the downregulation of the TF-encoding gene (KLF5, ASCL1 and TCF7; figure 4; electronic supplementary material, table S11).

### (d) Putative enhancer regions associated with sulfide-responsive genes

To identify putative enhancer regions, we first filtered all csRNA-seq peaks based on annotations consistent with expectations of enhancer RNAs (distal from the promoters of annotated genes and comprising unstable transcripts). This resulted in 33 799 regions, 448 of which were found to be significantly differentially initiated between sulfidic and non-sulfidic ecotypes (FDR < 0.05); hereafter, these 448 peaks are referred to as differentially active putative enhancer regions.

**Figure 5.**
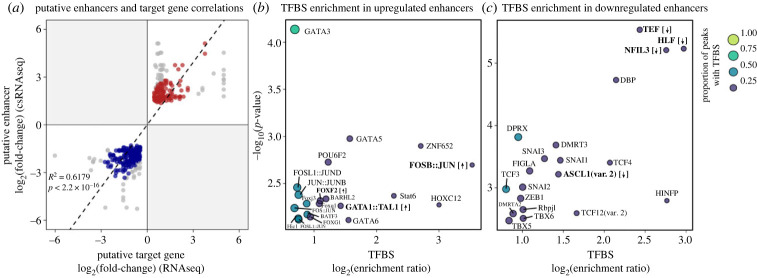
(*a*) Correlations between the log_2_ fold-change values of differentially active putative enhancer regions (*y*-axis) and potential differentially expressed target genes (*x*-axis). Red and blue points indicate H_2_S-upregulated and H_2_S-downregulated enhancer–gene pairs that fall within one standardized residual of the regression line, respectively. Grey points fall outside of one standardized residual of the regression line. (*b,c*) The top 20 most significantly enriched transcription factor binding sites (TFBSs) in (*b*) significantly upregulated putative enhancers (log_2_ fold-change > 0, FDR < 0.05) with high correlation to a nearby upregulated gene (red points in (*a*); log_2_ fold-change > 0, FDR < 0.05), and (*c*) significantly downregulated putative enhancers (log_2_ fold-change < 0, FDR < 0.05) with high correlation to a nearby downregulated gene (blue points in (*a*); log_2_ fold-change < 0, FDR < 0.05). The log_2_ enrichment ratio is represented on the *x*-axis, with points to the right in each panel having a higher degree of overrepresentation in the target peak set compared with the background. The −log_10_(*p*-value) of the enrichment test is represented on the *y*-axis, with points towards the top of each panel having lower *p*-values. Point size and colour are scaled by the proportion of target peaks possessing a given TFBS. Bold labels indicate TFBSs for which the gene encoding that TF is significantly upregulated (up arrows) or downregulated (down arrows) in the sulfidic ecotype. The '::' separates co-binding TFs. For a list of all enriched TFBSs, see electronic supplementary material, table S12.

Of the 2852 differentially expressed genes identified between ecotypes, 535 (18.8%) were located within 500 kbp of one or more differentially active putative enhancer regions. The majority of these genes (56%) were differentially expressed in the same direction as the nearby enhancer in the sulfidic ecotype. For this set of putative enhancer–gene pairs with the same direction of upregulation/activity in sulfidic fish, we correlated csRNA-seq and mRNA-seq log_2_ fold-change values and retained enhancer–gene pairs falling within one standardized residual of the linear regression line. This resulted in 213 enhancer–gene pairs, with 86 upregulated in the sulfidic ecotype (red points, figure 5*a*) and 127 downregulated (blue points, figure 5*a*; electronic supplementary material, table S12). Neither upregulated nor downregulated enhancer–gene pairs were enriched for candidate H_2_S detoxification genes (two-sided Fisher's exact test, *p* = 0.2307), although two upregulated candidate genes, *slc26a5* and *slc26a11*, were inferred to be targeted by upregulated putative enhancer regions.

We identified 32 and 31 TF binding sites that were significantly enriched in the parallel H_2_S-upregulated and H_2_S-downregulated enhancer–gene pairs, respectively (figure 5*b,c*; electronic supplementary material, table S13). Six genes coding for TFs enriched in the upregulated enhancers were themselves upregulated in the sulfidic ecotype based on gene expression data, while genes encoding three of the TFs enriched in the downregulated enhancer set exhibited downregulated gene expression (figure 5*b,c*; electronic supplementary material, table S14). In upregulated putative enhancer regions, the FOSB::JUN TF binding site was the most enriched relative to the background, and increased activity of this TF complex is further supported by the upregulation of the gene encoding *fosb* in the sulfidic ecotype (figure 5*b*). In downregulated putative enhancer regions, DBP, NFIL3, TEF and HLF TF binding sites were the most significant and highly enriched TFs compared with the background, with the latter three being further supported by downregulation of the TF-encoding gene (figure 5*c*).

## 4. Discussion

Examining regulatory mechanisms underlying adaptation to toxic levels of H_2_S in extremophile fishes allows us to gain a new functional understanding of some of the most highly conserved metabolic and detoxification pathways in metazoans. To achieve this, we quantified active transcription occurring outside of coding regions in sulfidic and non-sulfidic populations. We found convergent shifts in nascent transcription in sulfidic populations of *P. mexicana* (including *P. sulphuraria*) compared with non-sulfidic populations. Our results show that transcriptional differences between these ecotypes extend beyond that of mature, stable mRNA. As is true in other vertebrate systems, nascent transcripts likely reflect regulatory activity that affects gene expression [12,59]. Several differential transcription initiation sites mapped close to or within genes coding for enzymes involved in H_2_S detoxification (persulfide dioxygenase, sulfide : quinone oxidoreductase) and metabolism (glutathione *S*-transferase zeta 1). There was also differential transcription initiation in the promoter region of a solute carrier gene, s*lc16a6*, that is inhibited by sulfur-based compounds [60]. Within ecotypes, we found that nascent transcription patterns cluster samples by drainage, suggesting population-specific variation in transcription initiation and regulatory strategies that were not captured by ecotype comparisons alone. Drainage-specific physiological differences have been documented in the *P. mexicana* species complex previously [4,22], and examining regulatory differences that may underlie this physiology warrants future study with larger within-drainage sample sizes.

Most differential transcription initiation shifted in parallel with nearby gene expression, such that peaks with higher csRNA-seq signal in sulfidic populations were often near to genes with upregulated expression, and *vice versa*. We identified sets of enriched TF binding motifs in these differentially initiated csRNA-seq peaks occurring in the promoter-TSS of genes with parallel shifts in expression. Binding sites for several members of the Interferon regulatory factors (IRF) family of TFs were found to have the greatest magnitude of enrichment in the parallel upregulated promoter-TSS peaks. This family of TFs is typically involved in immune and pro-inflammatory responses to pathogens [61]. H_2_S has been shown previously to reduce activation of IRF3 and associated inflammatory responses to viral infection [62]. However, IRF3 was among the most enriched binding sites in parallel upregulated promoter-TSS peaks, and the gene encoding this TF was also found to be upregulated in the sulfidic ecotype. Members of the GATA family of TFs were overrepresented in upregulated promoter-TSS csRNA-seq peaks, including GATA1–3, involved in the haematopoietic system [63–65], and GATA4–6, involved in heart development [66]. While this may be due in part to nucleated blood present in the gill tissues at the time of sampling, the GATA TFs may have important roles in sulfide adaptation given that H_2_S binds haemoglobin, decreasing its effectiveness [67], and that erythropoiesis is regulated in response to hypoxia in fishes [68]. Two genes annotated in the *P. mexicana* genome as *GATA2* and a gene encoding TAL1, a TF that co-binds along with GATA1, are upregulated in the sulfidic ecotype, providing further evidence for the increased activity of these TFs in response to H_2_S.

Among the TF binding sites found to be enriched in H_2_S-downregulated promoter-TSS peaks, RUNX1 was present in a high proportion of peaks despite a relatively low enrichment ratio compared with the background sequences (figure 4*b*). RUNX1 interacts with hypoxia-inducible factor 1-alpha (HIF-1*α*), an important high-level regulator of cellular responses to hypoxia [69,70]. Additionally, these analyses identified enrichment of binding sites for ASCL1 (figure 4*b*), and the gene encoding ASCL1 was found to be H_2_S-downregulated in gene expression analyses. In amniotes, ASCL1 plays a critical role in the formation of pulmonary neuroendocrine cells that sense and initiate responses to hypoxia [71,72]. In other contexts (i.e. mammalian neuroblastomas), regulatory activity of ASCL1 has been shown to decrease in response to hypoxia [73]. While the exact roles and relevance of RUNX1 and ASCL1 activity in fish gills is not fully understood, their roles in sensing and responding to hypoxia in other systems suggests that they may play an important role in adaptation to sulfide springs with combined stress associated with H_2_S toxicity and hypoxia, warranting further investigation in future studies.

Our findings suggest that differential enhancer activity plays an important role in H_2_S adaptation by contributing to ecotype-specific gene regulation. Analysis of enriched TF binding sites in these putative enhancers revealed a suite of regulatory molecules that may stimulate shifts in gene expression in the sulfidic ecotype, many of which were not inferred to target the promoters of differentially expressed genes. Of note, the transcription factor FOXF2 was enriched in binding sites of H_2_S-upregulated putative enhancers targeting H_2_S-upregulated genes, and the *foxf2* gene was also upregulated in the sulfidic ecotype (figure 5*a*). In mammals, FOXF2 is highly expressed in the lung and is a major regulator of lung-specific gene expression [74,75]. Regulatory activity of FOXF2 has been shown to increase in response to cellular stress and hypoxic conditions [76]. Our inferences of increased FOXF2 regulatory activity in sulfidic populations of *P. mexicana* may suggest a similar role of FOXF2 in the response to hypoxia in fish gill, although subsequent study is needed to understand the precise role and relevance of this TF in H_2_S adaptation. In H_2_S-downregulated putative enhancers targeting H_2_S-downregulated genes, the transcription factors TEF, HLF, DBP and NFIL3 were among the most significantly enriched for binding sites (figure 5*b*). These four TFs belong to the PAR-domain basic leucine zipper TF family and are regulators of cellular responses to xenobiotics and metabolic detoxification [77,78]; accordingly, mice in which TEF, HLF and DBP are knocked out exhibit reduced expression and activity of downstream detoxification genes regulated by these TFs [77]. TEF, HLF and DBP have also been implicated in regulating pro-apoptotic responses to oxidative stress [79]. Evidence of decreased activity of these TFs in the sulfidic ecotype may indicate that aberrant activation of some detoxification pathways may be detrimental in H_2_S-adapted populations, similar to findings from studies of pollution-tolerant killifish populations [80]. However, subsequent experimental studies are needed to fully understand the relevance of these TFs in the sulfidic ecotype.

While the differential binding of TFs in promoter and enhancer regions likely contributes to many of the transcriptional differences separating the populations, relatively few genes encoding TFs with enriched binding sites were differentially expressed between ecotypes. It is probable that other mechanisms, such as differential splicing [81], phosphorylation (reviewed in [82]), sub-cellular localization [83], and dimerization (reviewed in [84]), have additional impacts on changes in TF function that were not examined here. Thus, the lack of differential gene expression for many TFs found to be enriched in promoter and/or putative enhancer peaks does not rule out their involvement or differential activity between ecotypes.

An important consideration of this study is that the *P. mexicana* reference genome assembly is highly fragmented (scaffold n50 = 275.3 kb, contig n50 = 39.8 kb), which limits our inference of genes targeted by putative enhancers to genes located on the same scaffold. This could result in the true target gene(s) of some putative enhancers going unidentified if not currently assembled on the same scaffold, and we therefore caution that our inferences of the genes targeted by differentially active putative enhancers remain preliminary at this stage. Should assembly contiguity improve in the future, the reanalysis of these data may reveal additional putative enhancer–gene pairs associated with H_2_S adaptation in this system. Further, the incorporation of other functional genomic evidence for enhancer activity using ATAC-seq and/or ChIP-seq would provide valuable clarity on both the differential activity and finer-scale TF binding of putative enhancers identified in this study.

In summary, our analyses revealed that transcriptional differences between the sulfidic and non-sulfidic ecotypes of the *P. mexicana* species complex differ beyond that of just gene expression and also include non-coding RNAs. Many genes differentially expressed between ecotypes also exhibited parallel shifts in nascent transcription in promoter, exonic and intronic regions. Additionally, analyses of putative enhancer regions suggest that differential enhancer activity contributes to ecotype-specific shifts in gene expression and provides a new and important set of genomic loci associated with H_2_S adaptation in this system. Ecotype-specific differences in TF activity inferred from TF binding site enrichment were apparent in both promoter and putative enhancer regions, implicating a large number of high-level regulatory molecules potentially driving responses to hypoxia and cellular stress in the sulfidic ecotype, and a subset of these TFs were further supported by the differential expression of the TF-encoding gene itself. Ultimately, our analyses showed clear ecotype-level differences in non-coding transcription and TF binding, both of which help explain changes in gene expression observed in the sulfidic extremophile populations of the *P. mexicana* species complex compared with non-sulfidic populations.

## Data Availability

Data are available from NCBI's GenBank. Accessions: PRJNA290391 (mRNA), PRJNA743555 (csRNA). Code for all analyses is available on Dryad: http://dx.doi.org/10.5061/dryad.83bk3jb0q [85]. Supplementary material is available online [86].
